# Human Rhinovirus 16 Causes Golgi Apparatus Fragmentation without Blocking Protein Secretion

**DOI:** 10.1128/JVI.01170-14

**Published:** 2014-10

**Authors:** Aurelie Mousnier, Dawid Swieboda, Anaïs Pinto, Anabel Guedán, Andrew V. Rogers, Ross Walton, Sebastian L. Johnston, Roberto Solari

**Affiliations:** Airway Disease Infection Section, National Heart and Lung Institute, Imperial College, London, United Kingdom

## Abstract

The replication of picornaviruses has been described to cause fragmentation of the Golgi apparatus that blocks the secretory pathway. The inhibition of major histocompatibility complex class I upregulation and cytokine, chemokine and interferon secretion may have important implications for host defense. Previous studies have shown that disruption of the secretory pathway can be replicated by expression of individual nonstructural proteins; however the situation with different serotypes of human rhinovirus (HRV) is unclear. The expression of 3A protein from HRV14 or HRV2 did not cause Golgi apparatus disruption or a block in secretion, whereas other studies showed that infection of cells with HRV1A did cause Golgi apparatus disruption which was replicated by the expression of 3A. HRV16 is the serotype most widely used in clinical HRV challenge studies; consequently, to address the issue of Golgi apparatus disruption for HRV16, we have systematically and quantitatively examined the effect of HRV16 on both Golgi apparatus fragmentation and protein secretion in HeLa cells. First, we expressed each individual nonstructural protein and examined their cellular localization and their disruption of endoplasmic reticulum and Golgi apparatus architecture. We quantified their effects on the secretory pathway by measuring secretion of the reporter protein Gaussia luciferase. Finally, we examined the same outcomes following infection of cells with live virus. We demonstrate that expression of HRV16 3A and 3AB and, to a lesser extent, 2B caused dispersal of the Golgi structure, and these three nonstructural proteins also inhibited protein secretion. The infection of cells with HRV16 also caused significant Golgi apparatus dispersal; however, this did not result in the inhibition of protein secretion.

**IMPORTANCE** The ability of replicating picornaviruses to influence the function of the secretory pathway has important implications for host defense. However, there appear to be differences between different members of the family and inconsistent results when comparing infection with live virus to expression of individual nonstructural proteins. We demonstrate that individual nonstructural HRV16 proteins, when expressed in HeLa cells, can both fragment the Golgi apparatus and block secretion, whereas viral infection fragments the Golgi apparatus without blocking secretion. This has major implications for how we interpret mechanistic evidence derived from the expression of single viral proteins.

## INTRODUCTION

Viral respiratory tract infections represent a major health care burden, with an estimated 500 million cases and a cost of $40 billion per year in the United States alone (1). It has been suspected since the 1970s that respiratory viral infections are a major trigger for asthma exacerbations in children and adults (2, 3), and with the development of more sensitive and specific diagnostics, it is clear now that around 80% of wheezing episodes in school-aged children and between half to three-quarters of wheezing in adults can be attributed to respiratory viral infections. Although there are a number of viruses implicated, rhinoviruses (HRVs) are the most frequently detected pathogens and are found in ∼65% of these cases (4–8). HRV infection also is known to be a major contributory factor in exacerbations of chronic obstructive pulmonary disease (COPD) (9–11).

HRVs are members of the positive-sense single-strand RNA Picornaviridae family, for which the best studied prototype is poliovirus (PV) and which includes other important pathogens, such as foot-and-mouth disease virus (FMDV), hepatitis A virus, and coxsackie virus (CV). Upon entering a host cell, the RNA genome of these viruses is translated into a polyprotein that is posttranslationally cleaved by the encoded viral proteases to generate the structural and nonstructural proteins required for viral replication. The developing virus forms replication complexes on the surface of intracellular membranes, believed to be derived either from the endoplasmic reticulum (ER)/Golgi secretory apparatus or from autophagosomes, and greatly remodels their morphology and lipid composition (12). For those viruses where it has been studied, the viral proteins 2B, 2C, and 3A and the intermediates 2BC and 3AB have been shown to be membrane associated, and these membranous replication complexes have been investigated intensely by microscopy, molecular, and biochemical studies (13, 14).

Although picornaviruses share a replication strategy, there appears to be significant and important differences between picornaviruses. The different techniques used to study them have not always generated consistent results. Picornavirus infection of host cells greatly remodels the morphology of the ER and Golgi apparatus, and some studies have shown that transfection and expression of the individual membrane-associated nonstructural proteins (NSPs) alone can replicate this effect. In some cases, viral replication or expression of 3A has been reported to cause an inhibition of protein traffic through the secretory pathway, but in some cases it does not. Whether a particular virus inhibits the secretory pathway is of great importance in host antiviral defense, as it would inhibit the secretion of inflammatory cytokines, antiviral interferons, and the cell surface expression of major histocompatibility complex (MHC) class I. Indeed, inhibition of the secretory pathway often has been cited as a mechanism by which picornaviruses evade host defense mechanisms.

Two independent studies showed that the 3A protein of CV and PV blocked secretion by binding and inhibiting the function of GBF1, the GTP exchange protein that controls the function of Arf1, whereas the 3A proteins of HRV2, HRV14, encephalomyocarditis virus, FMDV, and hepatitis A virus did not (15, 16). However, subsequent studies showed HRV1A infection and overexpression of the HRV1A 3A protein did cause Golgi structure fragmentation (17). Given these inconsistencies and the clinical importance of HRV, we considered it important to comprehensively investigate whether viral infection and expression of individual HRV16 nonstructural proteins did indeed affect the secretory pathway. We chose HRV16, as this is a well-characterized serotype and the most frequently used in human clinical studies in both asthma and COPD (18–21). To this end, we have transfected each individual nonstructural HRV16 protein into HeLa cells and determined their effect on morphological remodelling of the ER and Golgi apparatus. We also have quantified their effect on the secretory pathway by measuring the release of a Gaussia luciferase (Gluc) reporter. Finally, we compared these results to Golgi apparatus remodelling and luciferase secretion from HeLa cells infected with whole HRV16 live virus and demonstrate significant differences between these two techniques.

## MATERIALS AND METHODS

### Cell culture.

The cervical epithelial HeLa Ohio (ECACC 93021013) and HeLa H1 (ATCC CRL-1958) cell lines were maintained in exponential growth in high-glucose Dulbecco's modified Eagle medium (DMEM) supplemented with glutamine, 1% sodium bicarbonate, 25 mM HEPES, and 10% fetal bovine serum. The transfection of HeLa cells with mammalian expression plasmids was performed using FuGENE HD transfection reagent (Promega), with a 3.5:1 FuGENE HD-to-DNA ratio and doubling the volume of transfection mix added per well compared to the manufacturer's instructions. The HeLa Ohio cell line stably expressing Gluc was created by transfection with the pCMV-GLuc 2 plasmid (New England BioLabs) and selection with 1 mg/ml Geneticin-G418 (Invitrogen).

### Plasmid construction.

cDNA constructs for each HRV16 nonstructural protein (NSP) were made by PCR amplification using the following primer pairs (each NSP cDNA was cloned into pRK5-Myc [Clontech] using the restriction sites indicated below by standard molecular biology techniques): Myc-HRV162A, 5′-TATGATGGATCCGGGCCTAGTGACATGTATGTGC-3′ (BamHI) and 5′-GCGCGCGAATTCTCATTGTTCTTCAGCACAGTGAAAG-3′ (EcoRI); Myc-HRV16 2B, 5′-GACGACGGATCCGGAATCACTGATTACATACACATGC-3′ (BamHI) and 5′-CGCGCGAATTCTCATTCTTTGTGTATATAAGTTAATTGAG-3′ (EcoRI); Myc-HRV16 2C, 5′-GCGCGCGGATCCTCAGATTCATGGCTCAAAAAATTCAC-3′ (BamHI) and 5′-GCGCGCGAATTCTCATTGGAAAATTGCAGACATGACATC-3′ (EcoRI); Myc-HRV16 2BC, 5′-GACGACGGATCCGGAATCACTGATTACATACACATGC-3′ (BamHI) and 5′-GCGCGCGAATTCTCATTGGAAAATTGCAGACATGACATC-3′ (EcoRI); Myc-HRV16 3A, 5′-GACGACGGATCCGGGCCTATATCCATGGATAAACCC-3′ (BamHI) and 5′-GCGCGCGAATTCTCACTGTAGAGAGCAAAAGAGC-3′ (EcoRI); Myc-HRV16 3AB, 5′-GACGACGGATCCGGGCCTATATCCATGGATAAACCC-3′ (BamHI) and 5′-GCGCGCGAATTCTCATTGAGCTACCACTCTTCTCTCG-3′ (EcoRI); Myc-HRV16 3C, 5′-GAGTGCGGATCCGGTCCAGAAGAAGAATTTGGAATGTC-3′ (BamHI) and 5′-GCGCGCGAATTCTCATTGTTGTTCAGTGAAGTATGATCTC-3′ (EcoRI); Myc-HRV16 3D, 5′-GACGACGGATCCGGCCAAATTCAAATCTCTAAACATG-3′ (BamHI) and 5′-GCGCTCGCGATCAGAATTTTTCATACCATTCATGTCTTAG-3′ (NruI). All of the pRK5-Myc-HRV16 constructs contained the published HRV16 sequence (18) (GenBank accession no. L24917) and an N-terminal Myc tag. The sequence identity and correct orientation of all inserts were verified by DNA sequencing.

### HRV16 stock production and infections.

HRV16 viral stocks (ATCC VR-283) were produced by infecting HeLa H1 cells, and their titers were determined by measuring the 50% tissue culture infectious dose (TCID_50_) in HeLa Ohio cells.

HeLa Ohio cells were infected with HRV16 at a multiplicity of infection (MOI) of 1 or 20. Infections were synchronized by virus adsorption on the cells for 1 h at room temperature, followed by one wash with phosphate-buffered saline (PBS) and the addition of new media before incubating the cells at 37°C for 2 to 24 h, as indicated.

### Immunofluorescence microscopy.

Cells grown on glass coverslips were washed with PBS, fixed for 15 min with 4% formaldehyde, and washed with PBS. After quenching residual formaldehyde with 0.1 M glycine, cells were washed with PBS, permeabilized for 10 min at room temperature with 0.1% Triton X-100, and then washed with PBS. After blocking in 5% fetal bovine serum, cells sequentially were incubated with primary and secondary antibodies diluted in 1% bovine serum albumin (BSA). Apart from the calnexin antibody, which was incubated overnight at 4°C, primary antibodies were incubated for 1 h at room temperature and secondary antibodies were incubated for 45 min at room temperature. Cells were washed in PBS after each antibody incubation. Coverslips were mounted in ProLong Gold antifade reagent (Invitrogen) and analyzed using an LSM 5 PASCAL laser scanning microscope (Carl Zeiss).

The following primary antibodies were used at the indicated dilution: mouse anti-Myc tag (05-724; 1/500; Millipore), rabbit anti-calnexin (2679S; 1/50; New England BioLabs), rabbit anti-Giantin (ab80864; 1/500; Abcam), mouse anti-GM130 (610822; 1/500; BD Pharmingen), sheep anti-TGN46 (AHP500GT; 1/500; AbD Serotec), and rabbit anti-HRV16 2C (this study; 1/500). Secondary antibodies were obtained from Jackson ImmunoResearch (1/200).

### Electron microscopy.

One culture well of each HRV16-infected (3, 5, and 7 h) and uninfected control HeLa cells was scraped and fixed with 2.5% glutaraldehyde in 0.05 M sodium cacodylate buffer (pH 7.2). Cells were spun at 2,500 rpm (approximately 1,000 × *g*) for 5 min, resuspended in agar, and spun to form a pellet. The pelleted cells were rinsed in cacodylate buffer and postfixed in buffered 1% osmium tetroxide. After a water rinse, the samples were dehydrated by a graded (70 to 100%) methanol series. Sample transition to 100% araldite was through 50:50 and then 25:75 propylene oxide-araldite mixtures. Ultrathin (70-nm) sections of araldite-embedded samples were stained with uranyl acetate and lead citrate and then examined with a transmission electron microscope (Hitachi H7000).

### Flow cytometry.

HeLa Ohio cells were infected with HRV16 at an MOI of 20 for 7 h. After washing with PBS, cells were detached with Accutase (Life Technologies), washed a further time, and stained with a fixable near-IR live/dead discriminator (Life Technologies). Cells were fixed with 1% formaldehyde, washed, and incubated with 1% human serum (HS) overnight. Following permeabilization with 0.5% saponin-1% HS in PBS, cells were either stained with rabbit anti-HRV16 2C antibody (1/500; this study) or purified rabbit IgG control (1/500; R&D Systems). Cells were visualized with donkey anti-rabbit Alexa 488 secondary antibody (1/200; Jackson ImmunoResearch). Photomultiplier tube (PMT) voltages were adjusted after standardized cytometer setup and tracking (CST) checks minimizing the spectral overlap to increase data precision. Cells were measured on a Becton Dickinson Fortessa LSR-SORP equipped with various lasers (20 mW at 355 nm, 50 mW at 405 nm, 50 mW at 488 nm, 50 mW at 561 nm, and 20 mW at 633 nm) and an ND1.0 filter in front of the forward scatter (FSC) photodiode and analyzed with FlowJo software (TreeStar).

### Secretion assay.

For transfection experiments, HeLa Ohio cells were cotransfected in 24-well plates with pCMV-GLuc 2- and pRK5-Myc-derived plasmids at a ratio of 1:3 for 24 h. For infection experiments, HeLa Ohio cells stably expressing Gaussia luciferase (HeLa-Gluc) were plated at a density of 0.2 × 10^6^ cells per well in a 24-well plate, and each time point was performed in triplicate. Cells were infected with HRV16 at an MOI of 20 for 1 h at room temperature and then washed in PBS before adding fresh medium and starting a 7-h time course. During the infection time course, culture medium was removed and replaced every 30 min and cells collected every 1 h. As controls, HeLa-Gluc cells either were not infected with virus or were not infected but treated with brefeldin A (BFA) at a final concentration of 5 μg/ml. To determine the Gluc activity in the cells, the wells were washed with PBS and the cells lysed in 250 μl of luciferase cell lysis buffer (B3321; New England BioLabs). Gluc secreted in the media and remaining in the cells was assayed using the BioLux Gaussia luciferase flex assay kit (E3308S; New England BioLabs) according to the manufacturer's instructions. Luminescence was analyzed on a FLUOstar OMEGA plate reader (BMG Labtech) as recommended. Each time point was determined in triplicate, and the experiment was performed independently three times.

### Western blotting.

Cells were lysed in Laemmli sample buffer, and their DNA content was measured with a NanoDrop. An equivalent amount of DNA was loaded for each sample, and proteins were separated by SDS-PAGE. After transfer to PVDF membranes, proteins were revealed with a rabbit anti-HRV16 3A antiserum (produced for this study), a rabbit anti-β-actin antibody (3662-100; Bio Vision), or a mouse anti-Myc tag antibody (05-724; Millipore). Secondary antibodies were obtained from Jackson ImmunoResearch.

### Generation of antibodies.

A 29-amino-acid peptide corresponding to the N terminus of HRV16 3A (GPISMDKPPPPAITDLLRSVRTPEVIKYC) was synthesized and coupled to the carrier protein PPD, which had previously been derivatized to the heterobifunctional cross-linker MBS (Cambridge Research Biochemicals, United Kingdom). The peptide-PPD conjugate was used to immunize two rabbits using standard protocols (Covalab, France). A full-length cDNA sequence for HRV16 2C with an N-terminal 6-His tag was codon optimized and cloned into the bacterial expression plasmid pET-26b (Novagen) using the NdeI/XhoI sites. The full-length 2C protein was expressed in Escherichia coli BL21(DE3) and purified from inclusion bodies. The identity of the purified protein was confirmed by peptide mass fingerprinting. Purified HRV16 2C protein was used to immunize two rabbits using standard protocols (Covalab, France). All antiserum titers were quantified by enzyme-linked immunosorbent assay (ELISA), and their specificity was tested by Western blotting.

## RESULTS

### Transfected HRV16 nonstructural protein 2B induces endoplasmic reticulum aggregates.

**FIG 1:**
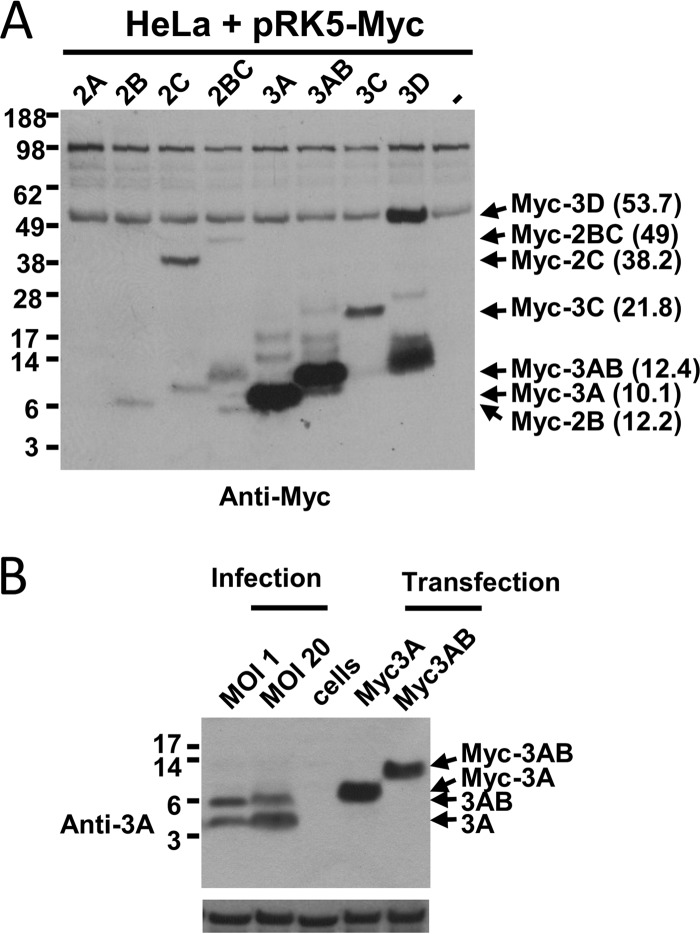
(A) Analysis of the ectopic expression of HRV16 nonstructural proteins by Western blotting. HeLa cells were transfected with the indicated pRK5-Myc constructs or the empty vector (−). Cells then were lysed in Laemmli sample buffer 24 h posttransfection, and the DNA content of the samples was assessed by NanoDrop measurement. The same amount of DNA was loaded for each sample, and the proteins were revealed by Western blotting with an anti-Myc antibody. The position of the proteins is indicated on the right, together with their predicted molecular mass in kDa. (B) HRV16 3A and 3AB are expressed at higher levels from transfected cells than from infected cells. HeLa Ohio cells were infected for 24 h with HRV16 at the indicated MOI, transfected with cDNA constructs allowing the expression of N-terminal Myc tagged HRV16 3A or 3AB, or left untreated (cells). Cells then were lysed in Laemmli sample buffer, and the DNA content of the samples was assessed by NanoDrop measurement. The same amount of DNA was loaded for each sample, and the 3A protein and β-actin loading control were revealed by Western blotting with anti-3A and anti-β-actin antibodies, respectively.

In order to assess the subcellular localization of the HRV16 nonstructural proteins, each individual protein was expressed in HeLa cells with an N-terminal Myc tag by transfection. The expression of the proteins was verified by Western blotting with an anti-Myc antibody, revealing the expression of all HRV16 nonstructural proteins apart from 2A (Fig. 1A). Transfection with 2A appeared to induce substantial cell death; consequently, protein expression probably fell below the detection limit. Expression of 3D produced the expected 54-kDa protein, but a 14-kDa N-terminal fragment also clearly was detected. The expression levels of each nonstructural protein varied widely despite their best efforts to transfect as efficiently as possible. Moreover, expression levels of 3A and 3AB were particularly high and even higher than their cellular levels following infection at an MOI of 1 or 20 (Fig. 1B).

**FIG 2:**
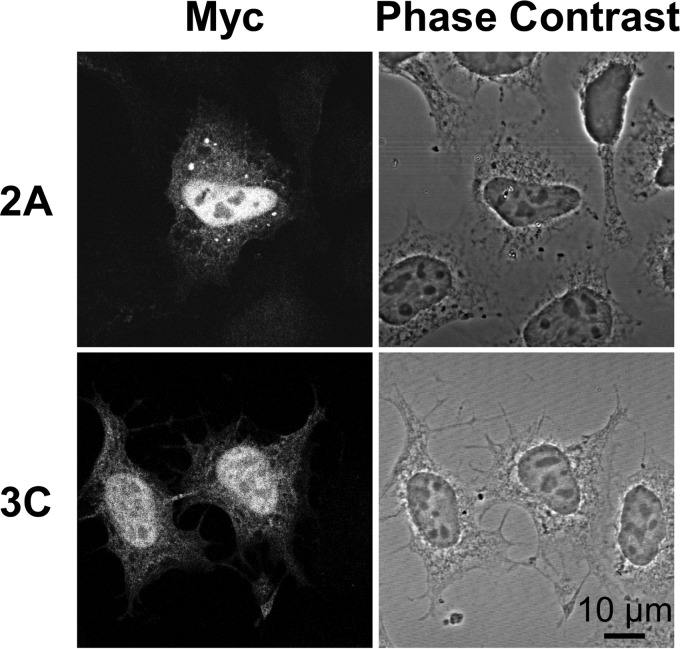
Localization of HRV16 proteases in transfected HeLa cells. HeLa Ohio cells were transfected with cDNA constructs allowing the expression of the HRV16 proteases 2A and 3C with an N-terminal Myc tag. Cells then were stained by immunofluorescence with an anti-Myc antibody.

**FIG 3:**
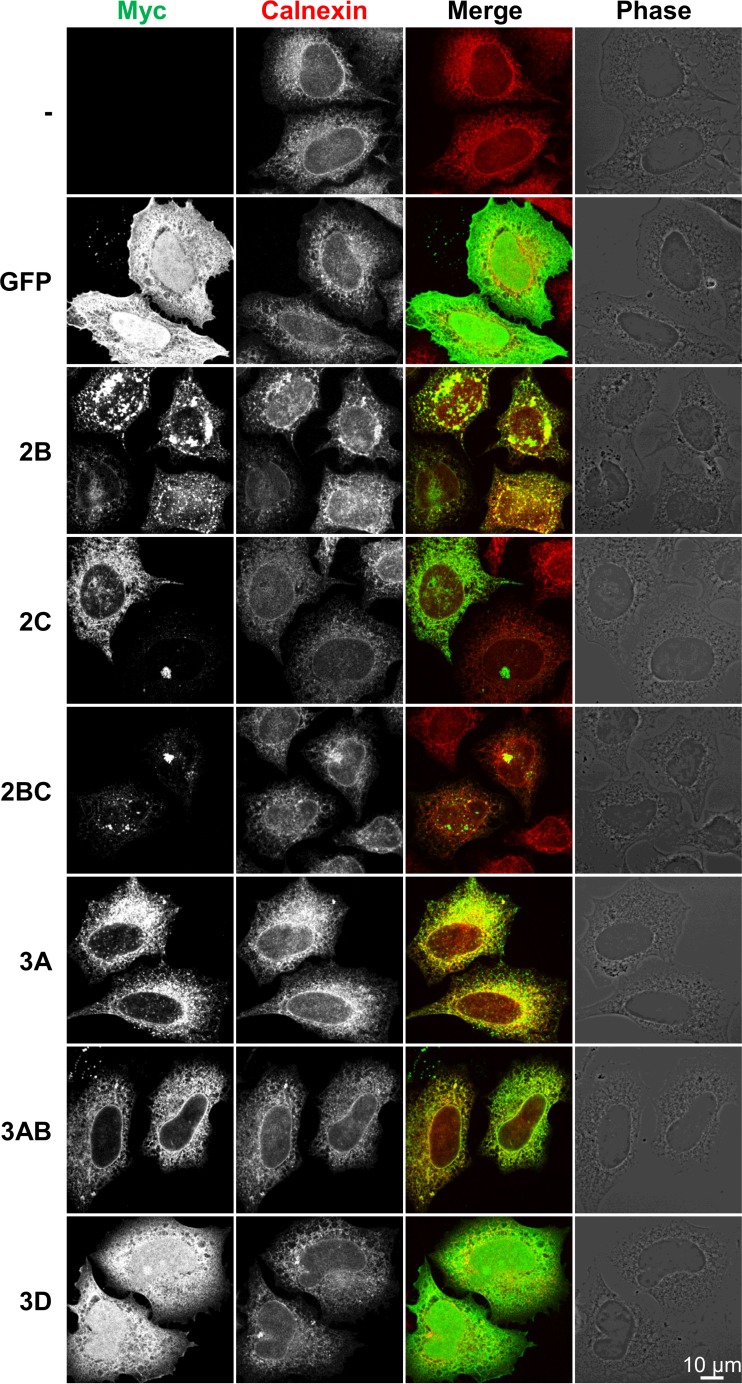
Effect of rhinovirus HRV16 nonstructural protein expression on the endoplasmic reticulum. HeLa Ohio cells were transfected with cDNA constructs allowing the expression of the indicated HRV16 nonstructural proteins or the GFP control with an N-terminal Myc tag. The nonstructural proteins and the endoplasmic reticulum marker calnexin were stained by immunofluorescence with anti-Myc and anti-calnexin antibodies, respectively, revealing partial colocalization of the nonstructural proteins 2C, 3A, and 3AB with calnexin and redistribution of the endoplasmic reticulum marker to 2B- and 2BC-containing aggregates. Images are single confocal 0.37-μm optical sections. Scale bar, 10 μm.

The analysis of the transfected cells by immunofluorescence microscopy with an anti-Myc antibody revealed that the viral nonstructural proteins 2A, 3C, and 3D had a diffuse localization in the cytoplasm and the nucleus, with 2A and 3C accumulating particularly in the nucleus of transfected cells (Fig. 2 and 3). However, contrary to the viral polymerase 3D, very few cells expressed the viral proteases 2A and 3C, and most cells detached from the coverslips, suggesting that the expression of 2A or 3C was cytopathic. Therefore, 2A and 3C were not investigated further.

The nonstructural proteins 2B, 2C, 2BC, 3A, and 3AB had cytoplasmic localization and colocalized to various extents with the ER marker calnexin (Fig. 3). In particular, 2B had a reticular distribution but also formed large aggregates and fine vesicles in the cytoplasm, both of which colocalized with calnexin staining, indicating that 2B localized to the ER and was able to redistribute it from a reticular pattern in control cells (transfected with the empty vector or the GFP cDNA) to 2B-stained aggregates. 2C displayed a reticular localization but also formed vesicles, often but not always located near the nuclear envelope. The reticular distribution of 2C partially colocalized with the ER marker calnexin. As for 2B, the 2BC protein also displayed a reticular distribution in addition to aggregates, which partially colocalized with calnexin. 3A presented a reticular and nuclear envelope staining which also colocalized with calnexin, in addition to a more punctate staining distributed throughout the cell cytoplasm which did not colocalize with calnexin. The 3AB precursor, on the other hand, only showed an ER-matching reticular and nuclear envelope staining.

### Transfected HRV16 nonstructural protein 3A and 3AB disrupt the Golgi structure.

**FIG 4:**
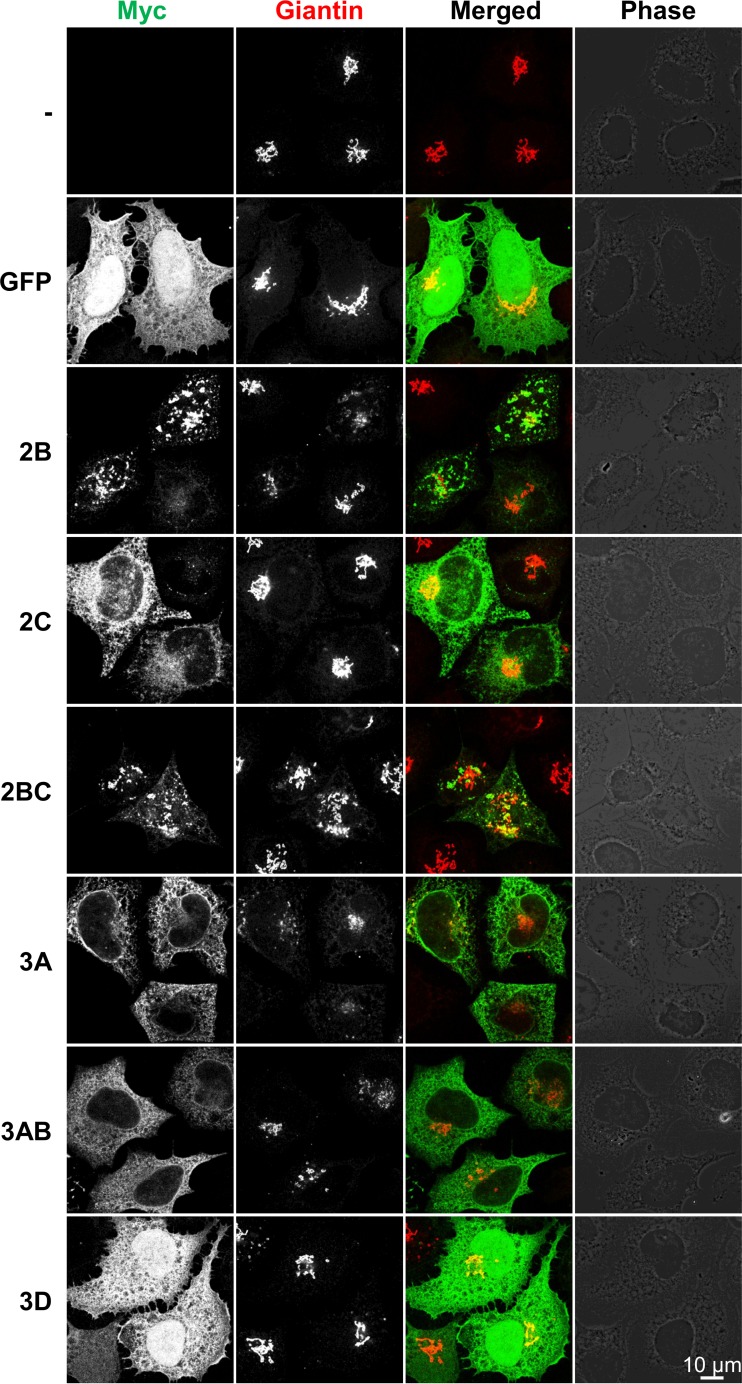
Effect of rhinovirus HRV16 nonstructural protein expression on the Golgi structure. HeLa Ohio cells were transfected with cDNA constructs allowing the expression of the indicated HRV16 nonstructural proteins or the GFP control with an N-terminal Myc tag. The nonstructural proteins and the Golgi marker Giantin were stained by immunofluorescence with anti-Myc and anti-Giantin antibodies, respectively, revealing some Golgi structure disruption upon expression of the nonstructural proteins 3A, 3AB, and, to a lesser extent, 2B compared to the compact Golgi structure observed in GFP-transfected cells. Images are single confocal 0.37-μm optical sections. Scale bar, 10 μm.

**FIG 5:**
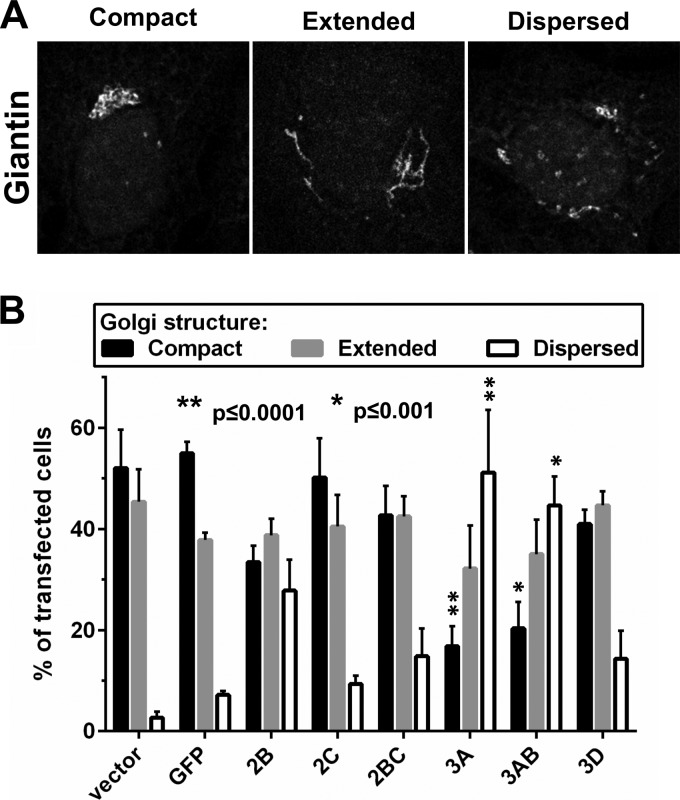
Transfected HRV16 3A and 3AB significantly disrupt the Golgi structure. HeLa Ohio cells were transfected with cDNA constructs allowing the expression of the indicated HRV16 nonstructural proteins or the GFP control with an N-terminal Myc tag. The nonstructural proteins and the Golgi marker Giantin were stained by immunofluorescence with anti-Myc and anti-Giantin antibodies, respectively. For each condition, the Golgi structure of 100 transfected cells was assessed and classified in one of the three categories depicted in panel A: compact, extended, and dispersed. For control vector-transfected cells, 100 random cells were evaluated. The mean number of cells in each category from 3 independent experiments is represented in panel B. Error bars represent the standard errors of the means (SEM). Results were analyzed with GraphPad Prism 6 software, using a two-way ANOVA followed by Dunnett's multiple-comparison posttest. Significant differences compared to the GFP-transfected control are marked.

To test if the HRV16 nonstructural proteins colocalized with or had an effect on the Golgi apparatus architecture, HeLa cells transfected with the Myc-tagged HRV16 nonstructural protein constructs were costained for the *cis*- and *medial*-Golgi marker Giantin, together with the Myc tag (Fig. 4). Strikingly, the Giantin distribution pattern was strongly disrupted upon 3A expression, from a compact localization in control cells transfected with GFP or the empty vector to a much more dispersed localization with a reticular distribution resembling that of typical ER staining. Together with the colocalization with the ER marker calnexin, this indicates that HRV16 3A disrupts the Golgi structure, probably by interfering with vesicular trafficking between the ER and the Golgi apparatus, thereby redistributing the Golgi components into the ER compartment. Some Golgi apparatus dispersion also was observed in 3AB-transfected cells, although this effect was less pronounced than that with the 3A construct, suggesting that the 3B fusion interferes with the ability of 3A to affect trafficking. Finally, 2B also mildly disrupted the Golgi structure in some of the transfected cells. Since Golgi structure can be variable in individual cells, we carefully quantified the Golgi dispersal phenotype. In 3 independent experiments, we confirmed that expression of 3A and, to a lesser extent, 3AB caused significant dispersal of the Golgi components (*P* < 0.0001 and *P* < 0.001, respectively), whereas the effect of 2B expression did not reach significance (Fig. 5). The expression of 2C, 2BC, and 3D did not significantly affect the Golgi structure (Fig. 4 and 5).

### Transfected HRV16 nonstructural proteins 2B, 3A, and 3AB inhibit cellular secretion.

**FIG 6:**
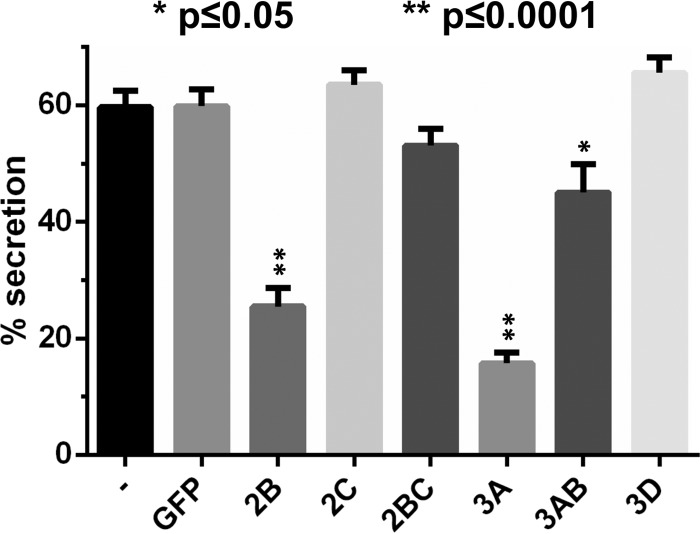
Transfected HRV16 2B and 3A significantly disrupt secretion of the Gaussia luciferase reporter. HeLa Ohio cells were cotransfected with constructs allowing the expression of the naturally secreted Gaussia luciferase (Gluc) and the indicated HRV16 nonstructural proteins or the GFP control with an N-terminal Myc tag. After 24 h of transfection, the cell medium was replaced and the Gluc was allowed to be secreted for 2 h. The Gluc activity then was measured in the medium and cell lysates, allowing the calculation of the percentage of secreted Gluc, which represents the Gluc activity in the media divided by the total Gluc activity present in the media and lysates combined. The data represent the mean percentages of Gluc secreted from 3 independent experiments. Error bars represent the standard errors of the means (SEM). Results were analyzed with GraphPad Prism 6 software, using one-way ANOVA followed by Dunnett's multiple-comparison posttest. Significant differences compared to the GFP-transfected control are marked.

As 2B induced ER aggregates and 3A and 3AB induced Golgi apparatus disruption, we evaluated if these events had any consequences on cellular protein secretion. We analyzed the secretion of a reporter protein, Gluc, from the copepod Gaussia princeps, which contains a native signal peptide at the N terminus that allows it to be trafficked through the classical secretory pathway (Fig. 6). A Gluc cDNA expression plasmid was cotransfected in HeLa cells together with the HRV16 nonstructural protein cDNA constructs. To ensure Gluc-expressing cells also expressed the transfected HRV16 NSP, cells were cotransfected with a mixture of 25% pCMV-Gluc and 75% pRK5-Myc-NSP plasmids. Twenty-four h after transfection, Gluc secretion over a 2-h period was analyzed by measuring Gluc activity in the media and the cell lysates. The percentage of Gluc secretion over 2 h, which represents the Gluc activity in the media divided by the total Gluc activity found in the media and cell lysates, was significantly inhibited in 2B- and 3A-transfected cells and, to a lesser extent, in 3AB-transfected cells compared to control cells transfected with GFP or the empty vector. The inhibition by 2B was significant despite relatively low levels of expression of the protein (Fig. 1A). This result indicates that 2B, 3A, and, to some degree, 3AB disrupts cellular protein secretion, in line with the observation that these proteins affect the ER or Golgi structure. The expression of 2C, 2BC, and 3D did not significantly affect Gluc secretion, although there was a trend for inhibition by 2BC. However, for 2BC, we cannot exclude that the small effect on Gluc secretion is due to its low expression levels (Fig. 1A).

### HRV16 infection disrupts the Golgi structure.

**FIG 7:**
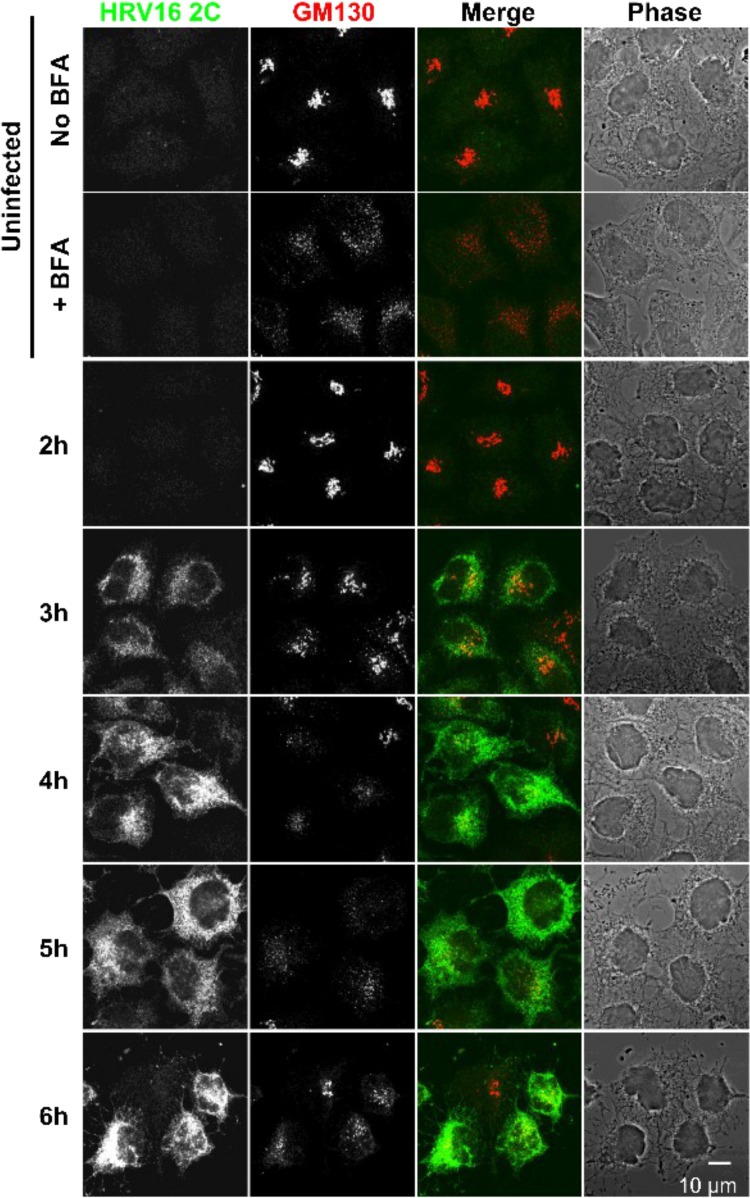
Effect of HRV16 infection on the *cis*-Golgi structure. HeLa Ohio cells were uninfected or infected with HRV16 at an MOI of 20 for the indicated periods of time. The nonstructural protein 2C and the Golgi marker GM130 were stained by immunofluorescence, revealing Golgi structure disruption in infected (2C-positive) cells compared to the compact Golgi structure observed in uninfected cells. As a control, HeLa cells were left untreated or treated with BFA to disrupt the Golgi structure. Images are single confocal 0.37-μm optical sections. Scale bar, 10 μm.

**FIG 8:**
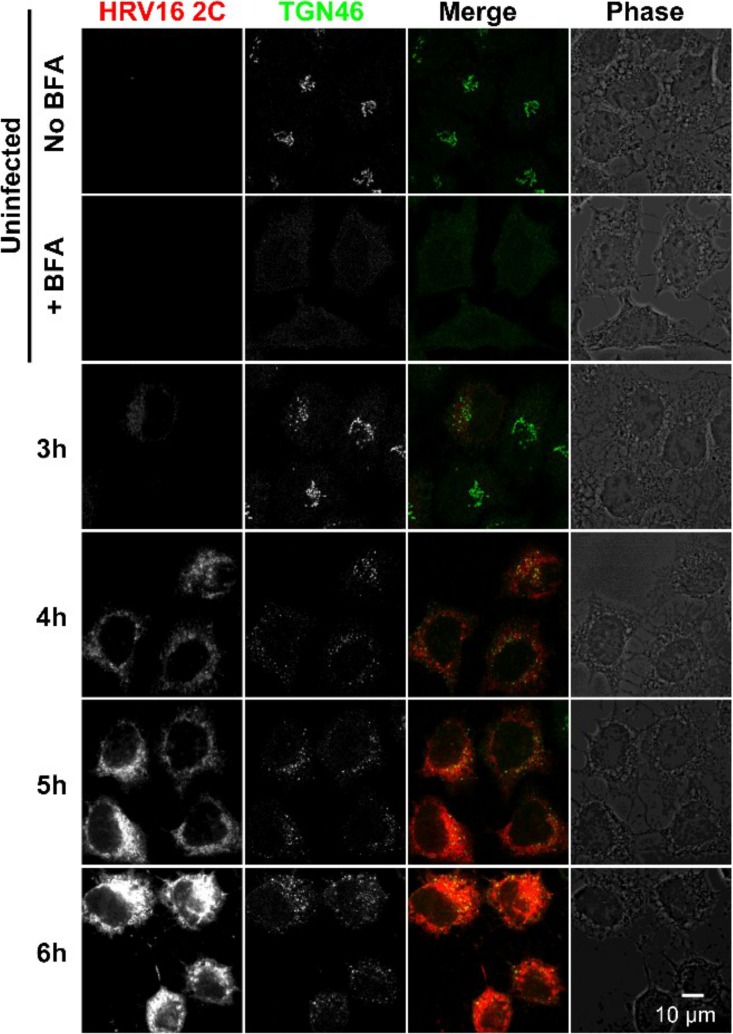
HRV16 infection disrupts the *trans*-Golgi network (TGN) structure. HeLa Ohio cells were uninfected or infected with HRV16 at an MOI of 20 for the indicated periods of time. The nonstructural protein 2C and the *trans*-Golgi network marker TGN46 were stained by immunofluorescence, revealing the disruption of the TGN structure in 2C-stained infected cells compared to the compact Golgi structure observed in uninfected cells. As a control, HeLa cells were left untreated or treated with BFA to disrupt the Golgi structure. Images are single confocal 0.37-μm optical sections. Scale bar, 10 μm.

**FIG 9:**
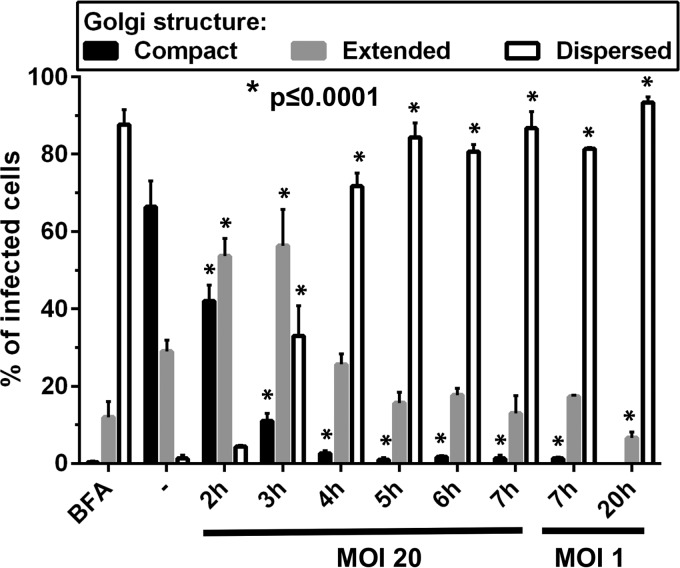
HRV16 infection significantly disrupts the Golgi structure. HeLa Ohio cells were uninfected or infected with HRV16 at the indicated multiplicity of infection (MOI) and for the indicated periods of time. Treatment with BFA was used as a control. The nonstructural protein 2C and the Golgi marker GM130 were stained by immunofluorescence. At a time point when infected cells could be detected by 2C staining, the Golgi structure of 100 infected (2C-positive) cells was assessed and classified in one of three categories, as depicted in Fig. 5A. Otherwise (for uninfected cells or cell infected for 2 h), 100 random cells were evaluated. Results represent the mean number of cells in each category from 3 independent experiments. Error bars represent the standard errors of the means (SEM). Results were analyzed with GraphPad Prism 6 software, using a two-way ANOVA followed by Dunnett's multiple-comparison posttest. Significant differences compared to the uninfected control are marked.

**FIG 10:**
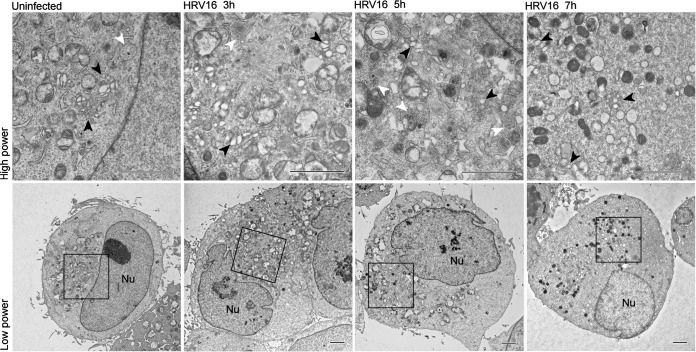
Electron microscopy of HRV16-infected HeLa cells. HeLa cells were infected with HRV16 at an MOI of 20 for 3, 5, or 7 h. In the uninfected cell, stacks of vesicular structures reminiscent of Golgi cisternae are apparent at high power (black arrowheads); however, in infected cells there are frequent swollen single-membrane vesicles (black arrowheads), and numerous multivesicular bodies (white arrowheads) are apparent in the cytoplasm. By 7 hpi, there are numerous electron-dense vesicular structures apparent which possibly are condensed mitochondria. Boxes represent the areas seen in the high-power-magnification images. The nucleus (Nu) is indicated at low power. Scale bars, 2.0 μm.

**FIG 11:**
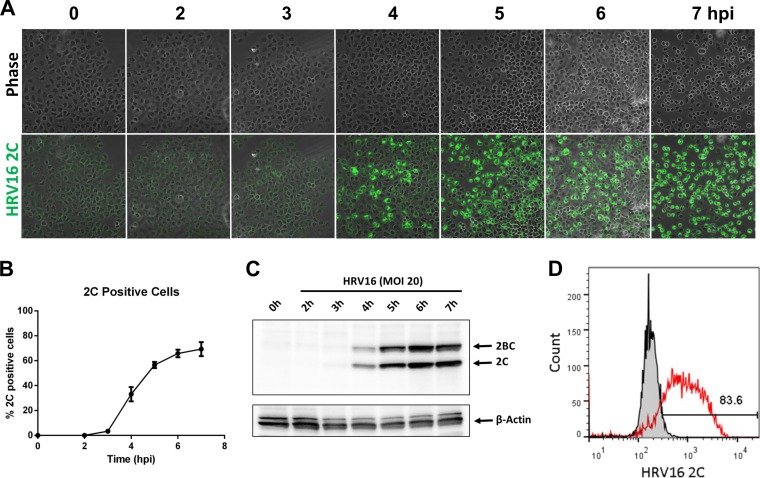
Time course of 2C expression following infection. HeLa cells were infected with HRV16 for various times. (A) 2C expression was detected by staining with an anti-2C antibody and immunofluorescence confocal microscopy. (B) Infected HeLa cells were analyzed by immunofluorescence confocal microscopy, and the percentage of cells staining positive for HRV16 2C was determined by manual cell counting. The results are the means ± SEM from 7 independent experiments. (C) HeLa cells were infected with HRV16, and cell lysates were prepared 0 to 7 hpi. Lysates were analyzed by SDS-PAGE followed by Western blotting, and 2C and 2BC were revealed by staining with an anti-2C antibody. The blot also was stained with anti-β-actin as a loading control. (D) HeLa cells at 7 hpi with HRV16 at an MOI of 20 were fixed and stained with anti-HRV16 2C and analyzed by flow cytometry. The gray filled curve represents a preimmune rabbit IgG control, and the red curve represents the cells stained with anti-HRV16 2C antibody. A total of 83.6% of the cells were gated as positive for 2C expression.

In order to evaluate the effect over time of HRV16 infection on the Golgi structure, we infected HeLa cells for different periods of time and monitored infection and the Golgi structure by immunofluorescence with an anti-HRV16 2C antibody, an anti-GM130 antibody, and an anti-TGN46 antibody, respectively (Fig. 7 and 8). As a control for Golgi structure dispersal, we treated cells with brefeldin A and stained them with anti-GM-130 or anti-TGN46. To have a maximal number of synchronously infected cells, an MOI of 20 was chosen. However, at this MOI, a noticeable cytopathic effect (CPE) could be observed between 6 and 7 h of infection. As a marker of viral replication, cells staining positive for 2C expression started to be observed from 3 h postinfection (see Fig. 11). From this time point, cells expressing the highest level of 2C already displayed some Golgi structure dispersion, compared to the uninfected control cells, for both the *cis*-Golgi marker (GM-130) and the *trans*-Golgi marker (TGN46). This effect increased as the infection time and 2C expression levels progressed, as evidenced by the quantification of the Golgi structure in 2C-positive infected cells from 3 independent experiments at MOIs of 20 and 1 (Fig. 9). These results indicate that HRV16 infection effectively disrupts the Golgi structure, leading to fragmentation of the Golgi structure into dispersed structures throughout the cell cytoplasm, with 86.7% of cells that were positive for 2C having dispersed Golgi structures at 7 hpi (MOI, 20) and 93.3% at 20 hpi (MOI, 1). This level of Golgi dispersal was quantitatively equivalent to treatment with BFA. We confirmed that the morphological disruption caused by viral replication by electron microscopy (Fig. 10). In infected cells, we observed frequent swollen single-membrane vesicles and multivesicular bodies and, by 7 hpi, the appearance of many electron-dense structures, probably condensed mitochondria.

### HRV16 infection does not block cellular protein secretion.

**FIG 12:**
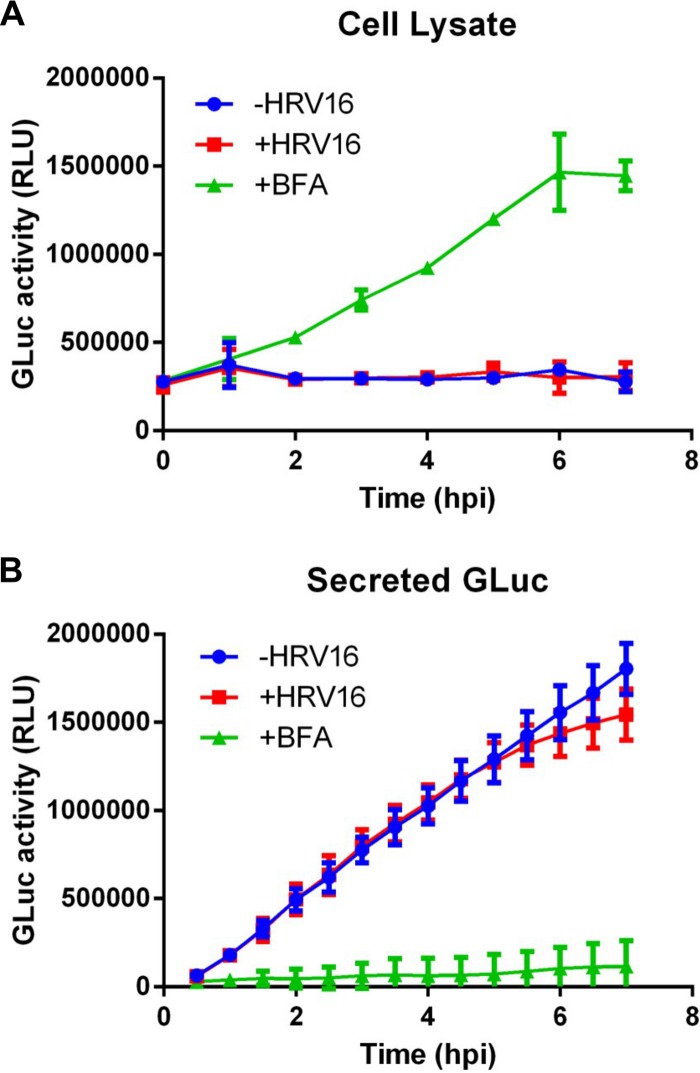
HRV16 infection does not block secretion of the Gaussia luciferase reporter. HeLa Ohio cells stably expressing the naturally secreted Gaussia luciferase (HeLa-Gluc) were infected with HRV16 (red squares) at an MOI of 20 for 1 h, followed by culture for the indicated periods of time up to 7 h postinfection (Time hpi). As controls, HeLa-Gluc cells were uninfected (blue circles) or uninfected but treated with BFA (green triangles). The cell culture medium was removed and replaced every 30 min, and the cells were harvested at 1-h intervals. The Gluc activity then was measured in the culture media and cell lysates. The cumulative secretion over time is calculated by adding each successive 30-min culture media sample to the previous total. Panel A shows Gluc activity in the cell lysate, and panel B shows secreted Gluc activity in the cell culture medium. Each point represents the mean relative light units (RLU) (± standard deviations) as a measure of Gluc activity from triplicate assay points from 3 independent experiments. Blue circles, uninfected HeLa-Gluc controls; red squares, HRV16-infected HeLa-Gluc cells; green triangles, BFA-treated HeLa-Gluc cells. The cumulative Gluc secretion into the culture medium from infected and uninfected cells was not significantly different at any time point (results were assessed with the GraphPad Prism 6 software using a two-way ANOVA with Bonferroni posttest analysis).

Since HRV16 infection induced Golgi structure dispersion, we analyzed if this functionally affected cellular protein secretion using the Gluc reporter secretion assay. As we found that transient transfection of cDNA plasmids had an inhibitory effect on infection, we established a HeLa cell line stably expressing Gluc (HeLa-Gluc) and used it for infection with HRV16 at an MOI of 20. We analyzed 2C expression following an infection time course as a marker for viral replication. HeLa cells were infected with HRV16, and 2C expression over a 7-h time course was detected by immunofluorescence confocal microscopy (Fig. 11A). The percentage of cells with detectable 2C expression by confocal microscopy was quantified by manually counting cells at various time points from 7 independent experiments. 2C was first detectable at 3 hpi and reached a maximum of 64.7% (±4.9 SEM; *n* = 7) by 7 hpi (Fig. 11B). The infection time course also was confirmed by Western blotting of extracts from infected cells and detection of 2C and 2BC with an anti-2C antibody (Fig. 11C). As an alternative technique, we quantified 2C-positive cells by flow cytometry and found that at 7 hpi, 83.6% of the cells had detectable 2C expression (Fig. 11D), suggesting that the determination of viral replication by immunofluorescence microscopy is an underestimate. To assess the impact of viral replication on protein secretion, we infected HeLa-Gluc cells at an MOI of 20 and collected the culture medium at 30-min intervals and cell lysates at 1-h intervals up to 7 hpi. The Gluc activity in the culture medium and cell lysates subsequently was measured. As controls, we measured levels of Gluc in cell lysates and culture medium from uninfected HeLa-Gluc cells and from cells treated with BFA. In parallel, we quantified 2C expression in cells by immunofluorescence with an anti-2C antibody to confirm infection. Over the 7-h time course, HRV16 replication did not significantly affect Gluc secretion compared to the uninfected control cells (when assessed by a two-way analysis of variance [ANOVA] with a Bonferroni posttest analysis), although between 6 and 7 hpi there was a trend to a reduced rate of Gluc secretion (Fig. 12). At 6 hpi, 64.0% of the cells stain positive for 2C (Fig. 11B) and 80.6% of 2C-positive cells have dispersed Golgi structures (Fig. 9); thus, at least 51.6% of the cells in the culture at this time point will have disrupted Golgi structures, although based on flow cytometry, this is likely to be an underestimate. Nevertheless, at 6 hpi there is no significant inhibition or reduction in Gluc secretion. Analysis of Gluc activity in the cell lysates clearly showed that levels of the marker remained constant over the 7-h time course, and that levels were comparable in infected and uninfected cells. Treatment of the HeLa-Gluc cell line with BFA clearly and completely inhibited Gluc secretion and resulted in Gluc accumulation in the cell lysate. Together, these results indicate that HRV16 infection leads to the dispersion of the Golgi but does not significantly block cellular protein secretion.

## DISCUSSION

In this study, we have sought to characterize the subcellular localization of individually transfected HRV16 proteins and their influence on the structure and function of the secretory pathway and to compare these effects to those of HRV16-infected cells.

We have shown that individual expression of HRV16 2B induced ER aggregates and slightly disrupted the Golgi structure, while expression of 3A and, to a lesser extent, 3AB, which also colocalized with the ER, induced significant Golgi structure dispersion. Expression of 2B, 3A, and, to a lesser extent, 3AB significantly inhibited cellular protein secretion, as evidenced by the reduction of the secretion of the Gluc reporter. The other nonstructural proteins did not significantly affect the ER or Golgi structure or Gluc secretion. These results show that inhibition of secretion is not directly related to the level of NSP expression (3A and 3AB being very high and 2B being very low) and not directly related to the degree of Golgi dispersal (3A and 3AB being significant and 2B less pronounced). Our results on the effect of HRV16 3A are consistent with the effect reported for HRV1A 3A (17), which is phylogenetically closely related to HRV16 (22). However, our data are clearly different from those of previous studies expressing 3A from HRV14 (15, 16, 23) and HRV2 (16) that showed that these proteins did not cause Golgi disruption or secretion inhibition. Similarly, studies on FMDV 3A showed that it too did not cause a secretion block (24). However, overexpression of 3A from other picornaviruses, such as PV (15, 16, 25–28) or CV (16, 23, 29), did block cellular protein secretion. The published evidence for a role for 2B and 2C in Golgi structure disruption also is conflicting. For CV, 2B and 2BC expression has been shown to cause a mild secretion inhibition without disrupting the Golgi structure (23) or a profound block in protein secretion (30). PV 2B has been shown to localize to what appeared to be intact Golgi structures but also caused a secretion block (25), whereas other studies reported Golgi structure disruption upon PV 2B expression (31). Some studies showed that PV 2C caused major remodelling of the Golgi structure (32) with no effect on protein secretion (25) or no Golgi structure association at all for FMDV 2C (33). The expression of FMDV 2BC or 2B and 2C together did block secretion (24, 34), and in a yeast model system (35), overexpression of 2BC caused a clear block in secretion and induced membrane proliferation. Finally, it has been shown recently that FMDV 3C is capable of causing Golgi fragmentation and blocking secretion (36). Therefore, the literature on the effect of picornavirus NSPs on the protein secretory pathway is abundant but not always consistent. The lack of consistency may be due to genuine differences between viruses, in expression of individual NSPs, the host cell line, and the type of assay or whether the NSP is tagged or not and perhaps even whether the tag is N or C terminal. In our experiments, we found highly variable levels of expression of the different NSPs when transfected into HeLa cells. 3A, 3AB, and 3D expressed at high levels, 2C and 3C to intermediate levels, 2B and 2BC expressed very poorly, and 2A was hardly detectable. However, despite its low level of expression, 2B caused a significant inhibition of protein secretion. Our studies comparing all of the NSPs in the same system show how difficult it is to make quantitative comparisons between experimental studies and possibly explains why the literature is so hard to reconcile on this topic. By comparison, there are relatively few studies looking at the block in protein secretion caused by viral infection and replication. There appears to be a complete block by PV (25) but only a partial block by CV (37), and there are several reports showing cytokine and chemokine secretion from CV-infected cells (38–40).

Given our concerns about studying the effects of individual NSPs on the secretory pathway, we decided to analyze the effect of HRV16 infection. We optimized an MOI and time course to give us as synchronous and uniform an infection as possible, and we found that over this time course viral replication led to a substantial dispersion of the Golgi structure but did not significantly block or reduce protein secretion. After 6 hpi, there was a trend for a reduction in the rate of Gluc secretion, although not a complete inhibition, and this corresponded with the onset of a CPE. The observation that infected and uninfected cells produced equivalent amounts of Gluc protein over the 7-h time course (medium plus cell lysate) suggests that there was not a noticeable translation shutoff of Gluc mRNA following infection. The Golgi structure fragmentation we observed is consistent with the many previously cited reports. It is well established that the infection of cells with picornaviruses induces dramatic rearrangements of membranes of the secretory pathway into double membrane-bound vesicles upon which viral replication complexes are thought to form, although the exact origin of these replication complexes still is unclear. Our electron microscopy (EM) studies with HRV16 do not reveal quite such dramatic membrane remodelling as that described for PV. We observed an increase in swollen single-membrane vesicles and multivesicular bodies and, by 7 hpi, frequent electron-dense structures of unknown origin but reminiscent of condensed mitochondria. There is evidence to suggest that PV and CV form replication complexes by modifying COPI vesicles through interference with GBF1/Arf1 (16, 41–46), and this is consistent with BFA inhibiting the replication of these picornaviruses. However, there are suggestions that replication complexes are derived from COPII-coated vesicles at ER exit sites (47, 48), and other evidence suggests they resemble autophagosomes (49, 50). However, neither COPII nor autophagosome formation is inhibited by BFA, which is known to be a potent inhibitor of PV, CV, and HRV replication, although not of all picornaviruses (51). Therefore, formation of picornavirus replication complexes is not clearly understood at present, and further investigations are required. Much effort has been made to identify interactions between picornaviruses and the host cell that can provide a mechanistic understanding of how the virus induces these structural changes and their significance for viral replication and the host antiviral response. Most work has focused on the membrane-associated viral proteins 2B, 2C, 3A, and their precursors 2BC and 3AB, often when transfected and expressed individually. A number of host proteins have been implicated in viral RNA translation and replication (52) and in formation of the replication complex, including Arf1 and its GTP exchanger GBF1 (13, 16, 41–43, 53), PI4KIIIβ (45, 54, 55), ACBD3 (54, 56), VCP (57), and OSBP (58, 59). Inhibitors of these pathways also are known to inhibit viral replication (55, 58, 60), confirming their importance and the role of the secretory pathway in the host-virus interaction. However, it appears that Golgi fragmentation is not the cause of the protein secretion block seen with PV, and it is proposed that these phenomena are independent of one another (61). Our data also show that for HRV16, Golgi fragmentation and secretion likely are independent phenomena.

The absence of a significant cellular protein secretion defect in infected cells is consistent with previously published reports on HRV-infected human bronchial epithelial cells and studies in patients showing that infected cells can secrete a wide range of cytokines, chemokines, and interferons and can upregulate MHC class I (62–70). Comparison between different HRV strains has shown that HRV16 and HRV1A are the most effective at inducing cytokine secretion (64). Although there is evidence for some picornaviruses that remodelling of the secretory pathway inhibits protein trafficking, our data, together with available evidence, suggest that HRV, unlike PV and CV, does not cause a secretion block. The ability of a virus to block cellular protein secretion or not has important implications for the host response to viral infection, as an inability to secrete inflammatory cytokines and interferons and to effectively traffic MHC class I to the plasma membrane would severely impair the host's ability to mount an effective antiviral response. For HRV, this is particularly important. Much effort is going into trying to understand why people with chronic respiratory diseases, such as asthma, COPD, or cystic fibrosis, appear to have an exaggerated response to this virus, and much of this effort is being directed at studying the cytokine, chemokine, and interferon responses of the epithelial cell to infection. Whether or not HRV blocks secretion from infected epithelial cells would be of major importance to our understanding of exacerbations of airway diseases and for our prospects of discovering effective remedies.

Our experiments with transfected HRV16 3A indicate that this nonstructural protein disrupts the Golgi structure and inhibits cellular protein secretion, whereas our infection experiments indicate that infection with the whole virus disrupts only the Golgi structure and does not inhibit cellular protein secretion. This suggests that studies based on the isolated expression of individual nonstructural picornaviral proteins are unlikely to reflect the complexity of the changes occurring during an infection, as proposed by others (61).

The observation that HRV16 infection leads to the dispersion of the Golgi structure but does not block cellular protein secretion raises several unresolved questions. First, picornaviruses disrupt the Golgi structure by a mechanism that is thought to act through the 3A protein interacting with GBF1, although it is not clear if this results in activation or inhibition of Arf1. BFA also fragments the Golgi structure by inhibiting Arf1, yet it is a potent inhibitor of viral replication (13, 16, 41, 71). What is the mechanistic significance of Golgi structure fragmentation in picornaviral replication? Moreover, from a cell biology perspective, the fact that HRV causes a dramatic fragmentation of the Golgi structure without blocking secretion is unexpected. Golgi structure fragmentation is observed in normal cells during mitosis (72) or as a result of DNA damage (73) through phosphorylation of GOLPH3, which is a PtdIns(4)P-binding protein. DNA damage also can induce senescence, and Golgi structure fragmentation also is observed in senescent cells (74). It is well known that the senescent phenotype is characterized by enhanced protein secretion (75). Thus, although Golgi fragmentation by HRV without blocking secretion is surprising, this is not an unprecedented biological phenomenon, and it confirms that there is still much to learn about how picornaviruses manipulate the secretory pathway.
